# Estimating the cost of wounds both nationally and regionally within the top 10 highest spenders

**DOI:** 10.1111/iwj.14709

**Published:** 2024-01-31

**Authors:** Douglas Queen, Keith Harding

**Affiliations:** ^1^ Medicalhelplines.com Inc Toronto Ontario Canada; ^2^ Cardiff University Cardiff UK

## 1 INTRODUCTION

Following our recent editorials regarding the estimation of the costs of wound care both globally and nationally,^1^ we published a few regional specific analyses.^2^, ^3^ To supplement this further, we carried out a similar analysis in the remaining top 10 highest spenders and updated their national costs to the most recent government data published. The regional analysis may vary by country depending on the collective statistics available for the regions.

The recent editorial introduced the approach to estimate the possible costs of wound care using freely available governmental health data, population statistics, and the research findings of many national groups.^1^ Using this methodology, an estimate, the costs of wounds nationally as a whole, and the individual regional elements of which the countries are comprised was carried out for those countries within the top 10 spenders, globally.

## 2 TOP 10 SPENDERS FOR WOUND CARE COSTS

The previous analysis^1^ focused on 2019 to permit a direct comparison between countries. The subsequent analyses focused on updating these figures to the most recently available estimate, based on the availability of regional governmental statistics. Also, in this analysis, we present the estimates in local currency, rather than international common currency. This will enable a broader understanding and provide an increased utility for researchers. This will be particularly useful for regional analysis within nations.

As with our previous analyses, the ‘accuracy’ of the estimate is dependent on what governmental information is available. One weakness is in some geographies that there is a lack of the availability of per capita healthcare spend on a regional basis. Perhaps within some geographies, the per capita healthcare spend is universal across all regions. To further our analysis, we made that assumption to permit a calculation of regional healthcare spend based on population statistics.

Irrespective of any inaccuracies in our estimation model, the data provides an indication of the likely regional spend across a geography, giving benchmark data for improvement initiative or governmental investment.

The following table provides a snapshot of the possible costs of wound care within these geographies in the year 2022.

Compared to our previous analysis,^1^ not unsurprisingly, the costs increased across all geographies. Interestingly, some minor changes in ranking within the top 10 did change.

## 3 REGIONAL ANALYSIS

Further analysis was carried out to provide a regional picture country by country. The results of this regional analysis are presented below.

### 3.1 Wound care costs–United States

The United States spends the most on healthcare globally.^4^ A national analysis (Table 1) suggests this is true for wound are also.^1^ The most recent governmental figures only permitted a regional analysis for 2020. Previous studies within the USA had provided an estimate of the likely costs,^5^, ^6^, ^7^, ^8^, ^9^ which are not outdated. None of these studies provided a regional analysis giving a picture of wound care spend state by state.

**TABLE 1:** Top 10 spenders in provision of wound care globally.

Country	2022 estimated wound care spend (Billion $PPP)	2022 estimated wound care spend (Local currency billions)
USA	$148.65	148.65 USD
China	$42.78	300.29 CNY
Japan	$22.91	2077.28 JPY
Germany	$23.33	16.79 EUR
France	$14.74	10.47 EUR
UK^3^	$13.31	8.82 GBP
Canada^2^	$9.34	11.13 CAD
Brazil	$11.18	28.84 BRL
Italy	$8.84	5.27 EUR
Australia	$6.04	8.66 AUD

**FIGURE 1:**
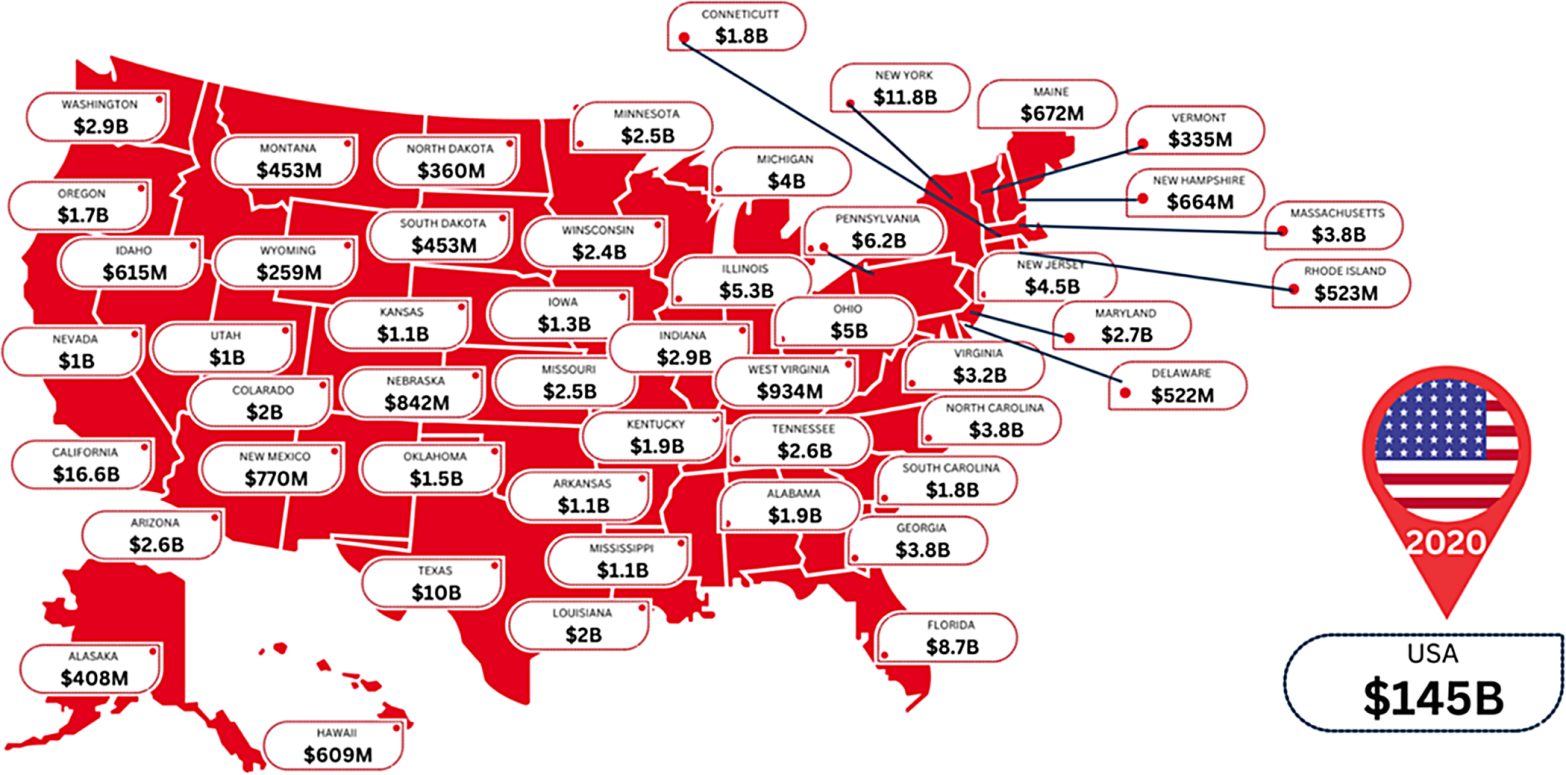
Estimated costs of wound care within the United States of America by state (2020).

The data presented in Figure 1 provide a crucial estimate of the likely costs of wounds across the United States. The costs are significant across all states, with some being more than many nations. These figures can provide a vital benchmark with regards to governmental/payor impacts both regionally and nationally.

### 3.2 Wound care costs–China

China may have lost the number one slot for total population recently, but our analysis estimates significant costs for wound care. A literature search highlighted a few studies with regards to wound care and its costs within China.^10^, ^11^, ^12^, ^13^

**FIGURE 2:**
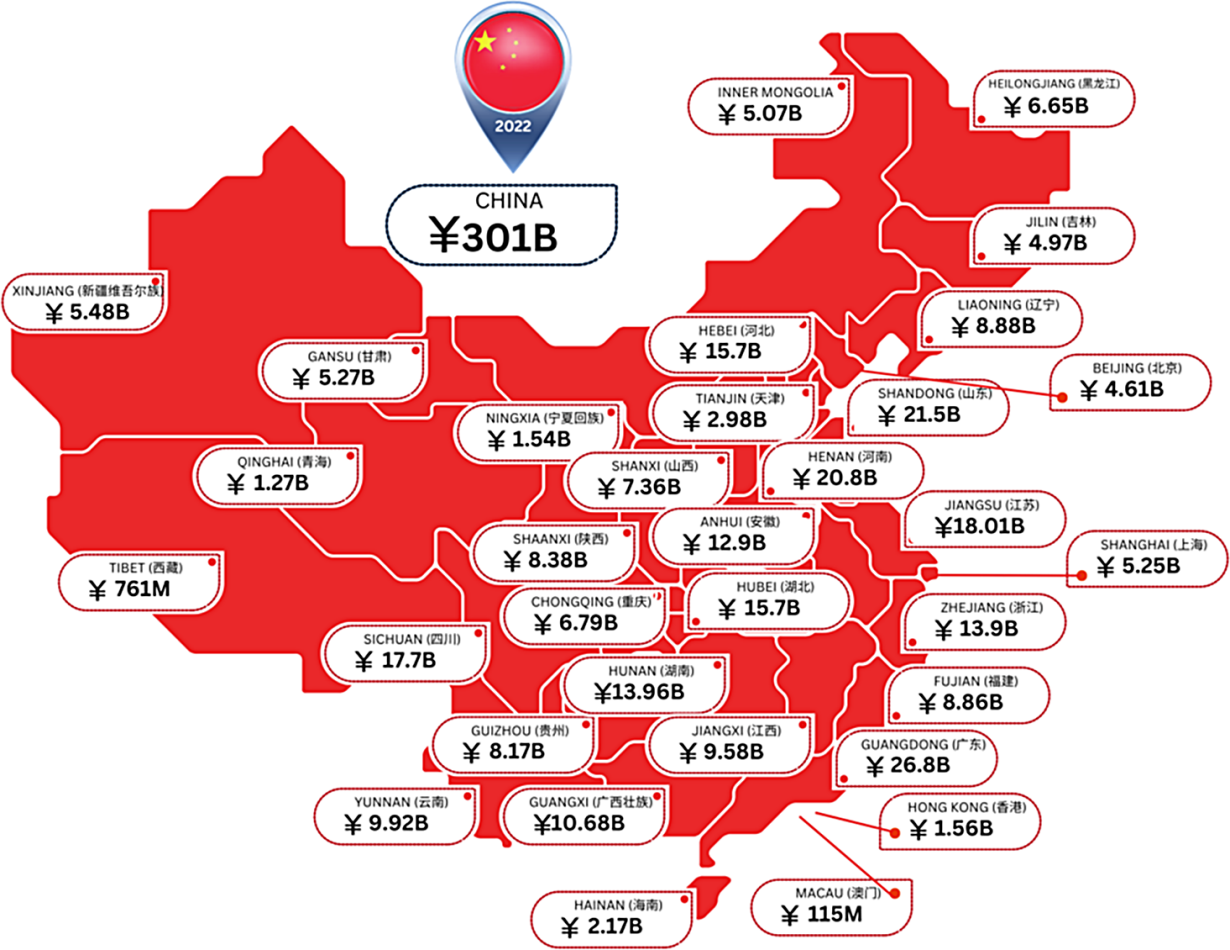
Estimated costs of wound care within China by region (2020).

A regional analysis shows for most of China's regions that their wound care costs equal that of many countries. This is not surprising since the estimation model is population based and China is developing economically with ongoing significant increases in per capita healthcare spend (Figure 2).

### 3.3 Wound care costs–Japan

Japan is one of the countries globally with a disproportionate elderly population. Since wound care is a problem for the elderly, then it should come as no surprise that Japan spends significantly in the wound care arena. Previous studies have shown this to be the case, especially in the pressure injury area.^14^, ^15^

**FIGURE 3:**
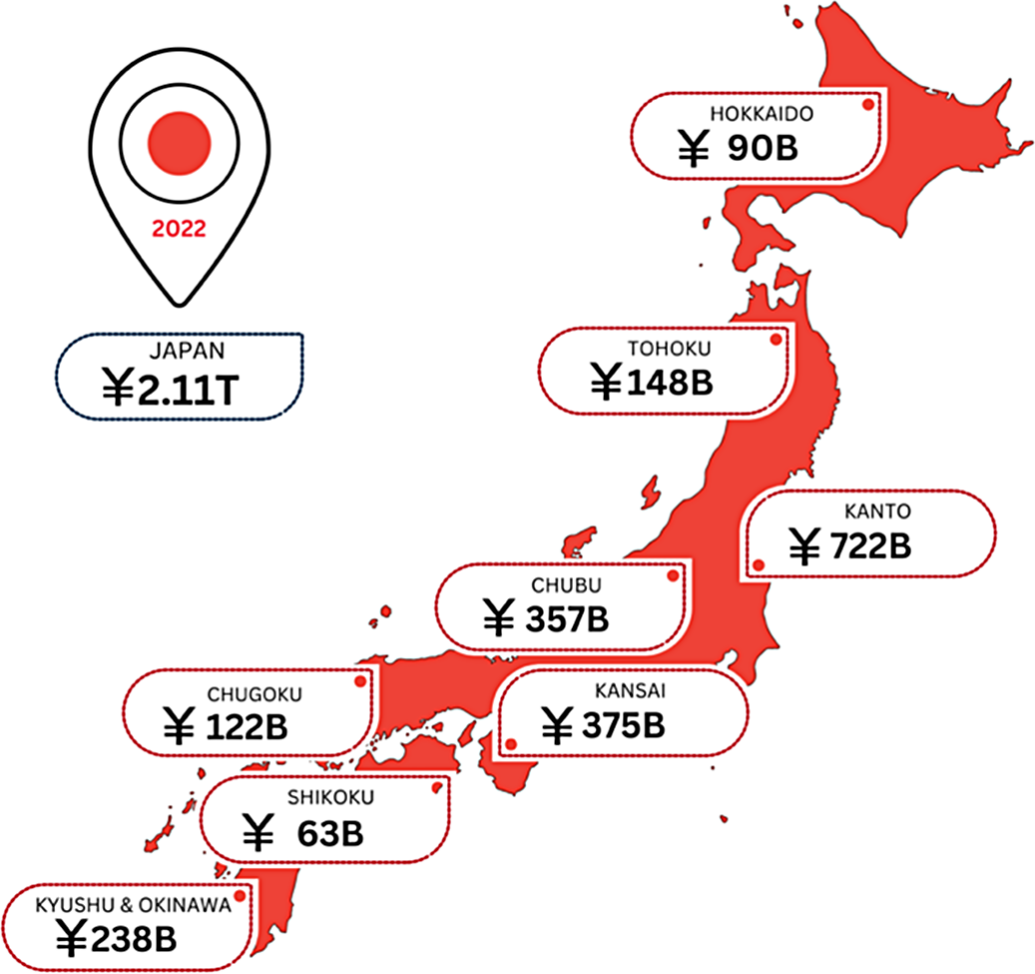
Estimated costs of wound care within Japan by region (2022).

Figure 3 presents the national and regional estimates of the wound care spend across Japan and its regions.

### 3.4 Wound care costs–Germany

Germany has a well‐structured healthcare system and a good handle on its healthcare costs. Several authors have published some costing studies within German.^16^, ^17^, ^18^, ^19^

**FIGURE 4:**
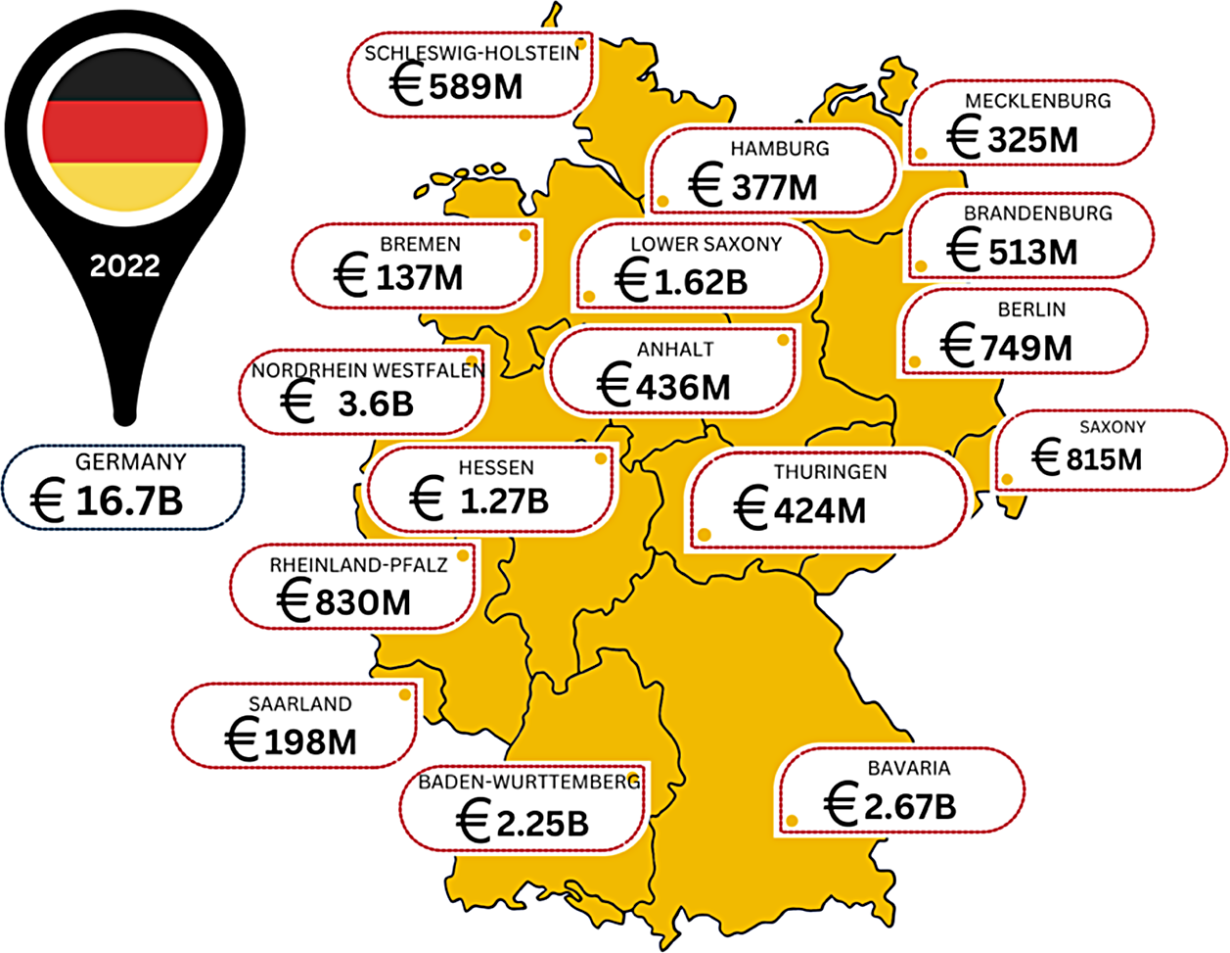
Estimated costs of wound care within Germany by region (2022).

Figure 4 provides a 2022 national estimate of cost, while providing a regional picture of the likely spend in the wound care area.

### 3.5 Wound care costs–France

**FIGURE 5:**
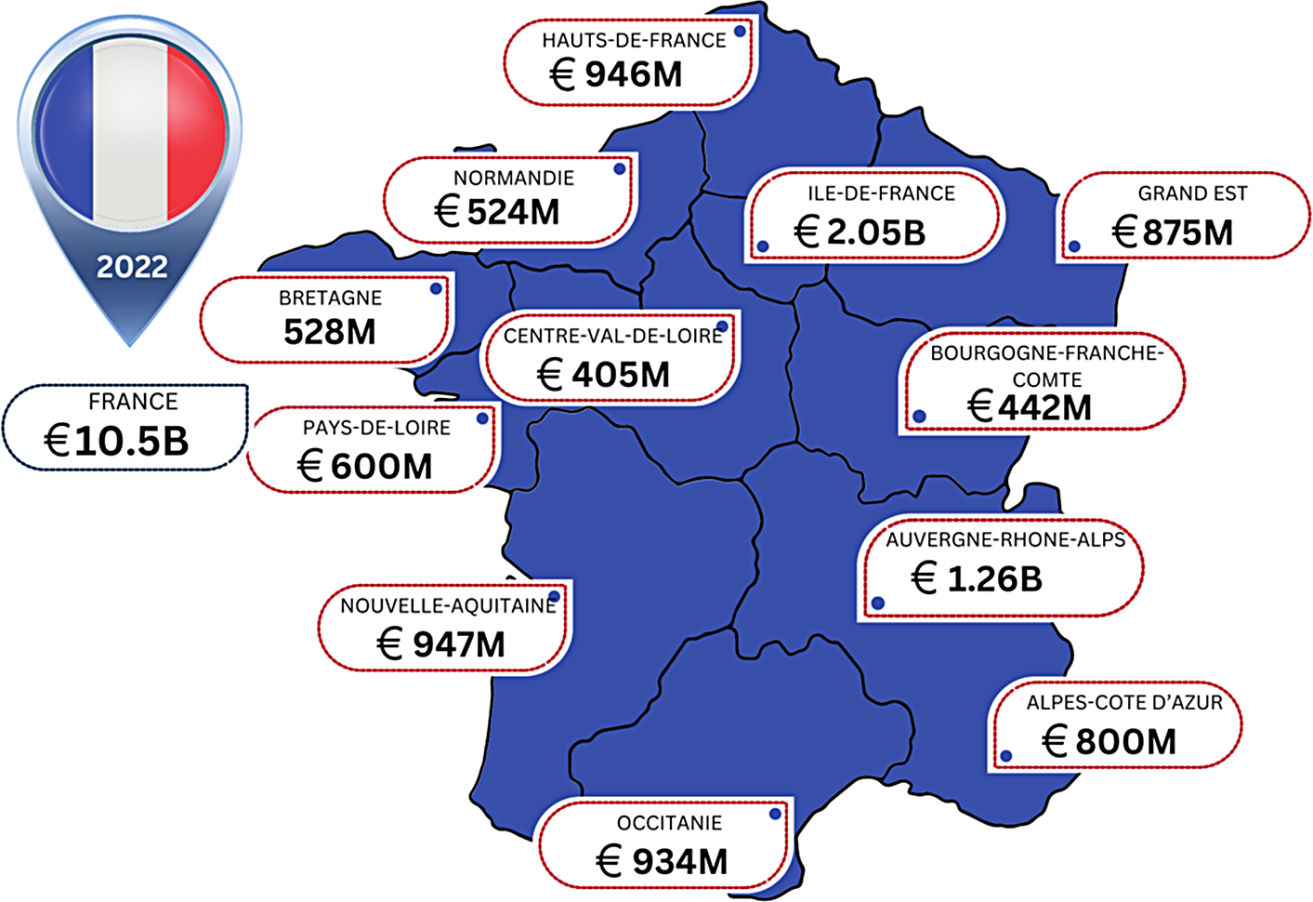
Estimated costs of wound care within France by region (2022).

France like Germany has a well‐developed and tracked healthcare system. A literature search shows there are few studies within France regarding the estimation of wound care costs.^20^ An analysis across France and its regions shows significant spend across the country (Figure 5).

### 3.6 Wound care costs–Brazil

Brazil is a quickly developing nation, with its healthcare systems becoming more and more sophisticated. With a large population its national healthcare spend is significant,^21^ and this includes wounds (Table 1). A literature search shows several studies have been carried out to highlight the cost of wounds within Brazil.^22^, ^23^ None, however, have provided a complete national cost nor indeed regional component.

**FIGURE 6:**
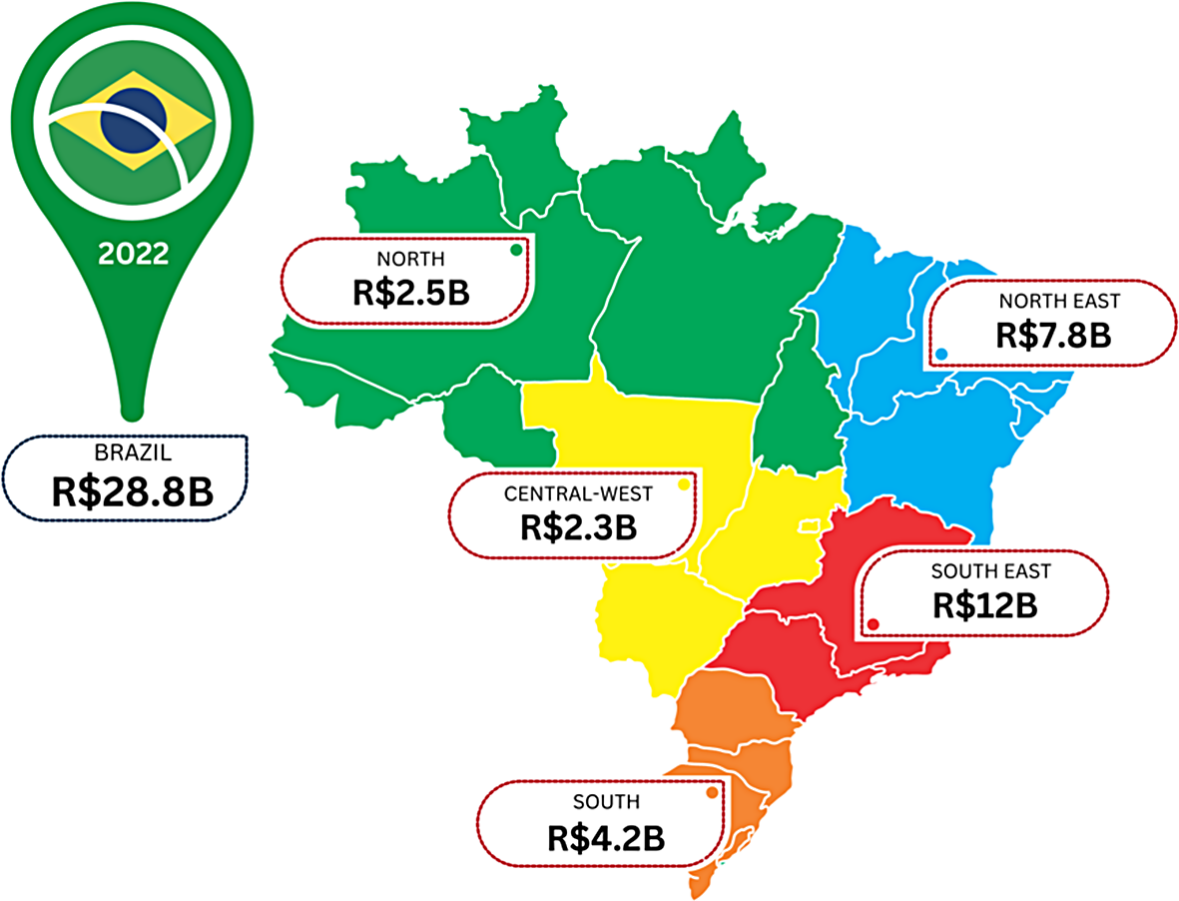
Estimated costs of wound care within Brazil by region (2022).

A regional analysis (Figure 6) demonstrates significant costs particularly in the east and south of the country.

### 3.7 Wound care costs–Italy

**FIGURE 7:**
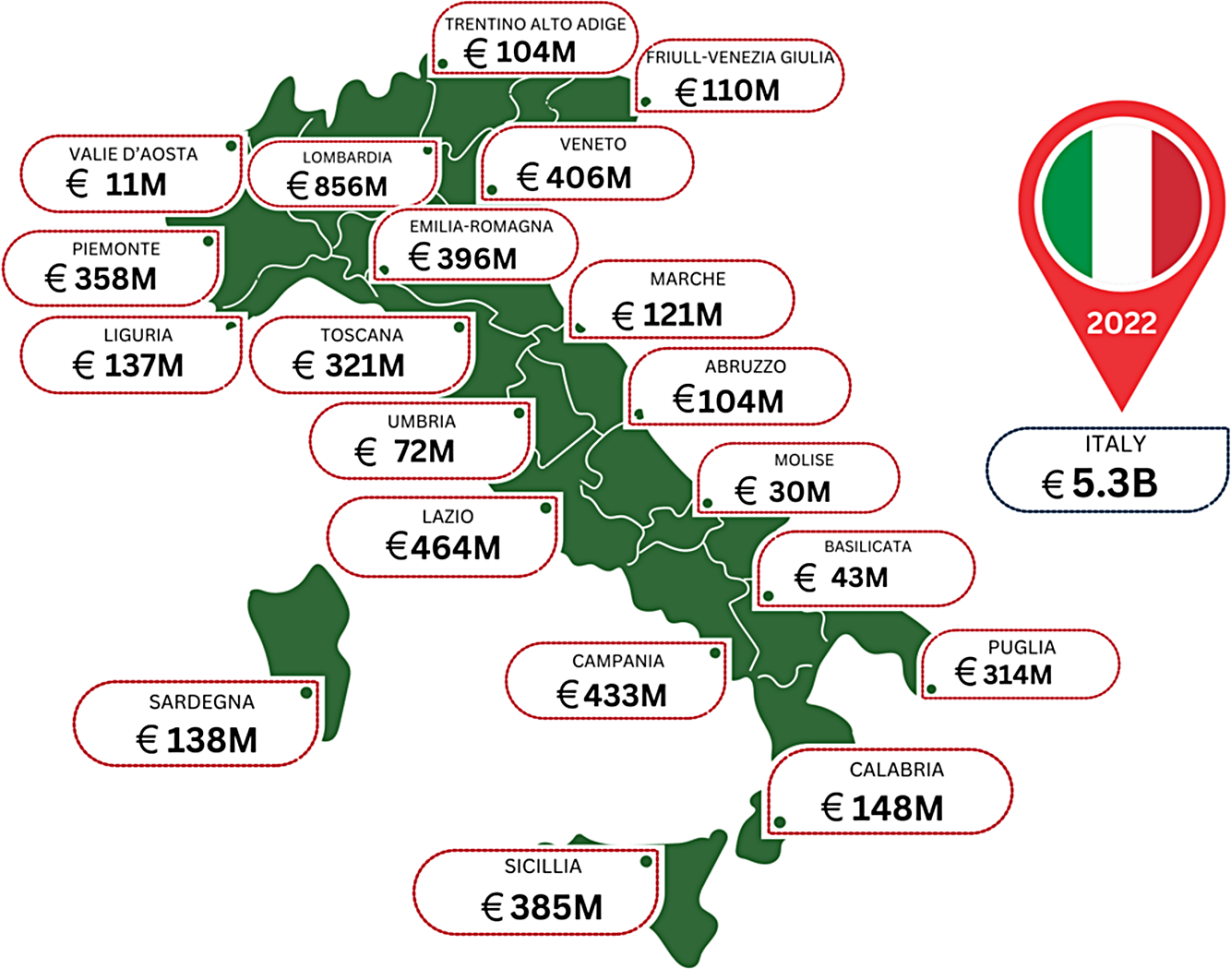
Estimated costs of wound care within Italy by region (2022).

Italy has one of the lower per capita healthcare spends within Europe.^24^ However, its estimated wound care spend is still significant as seen in Table 1 and supported by a previous study. A regional analysis presented in Figure 7 shows how this is broken down regionally.

### 3.8 Wound care costs–Australia

**FIGURE 8:**
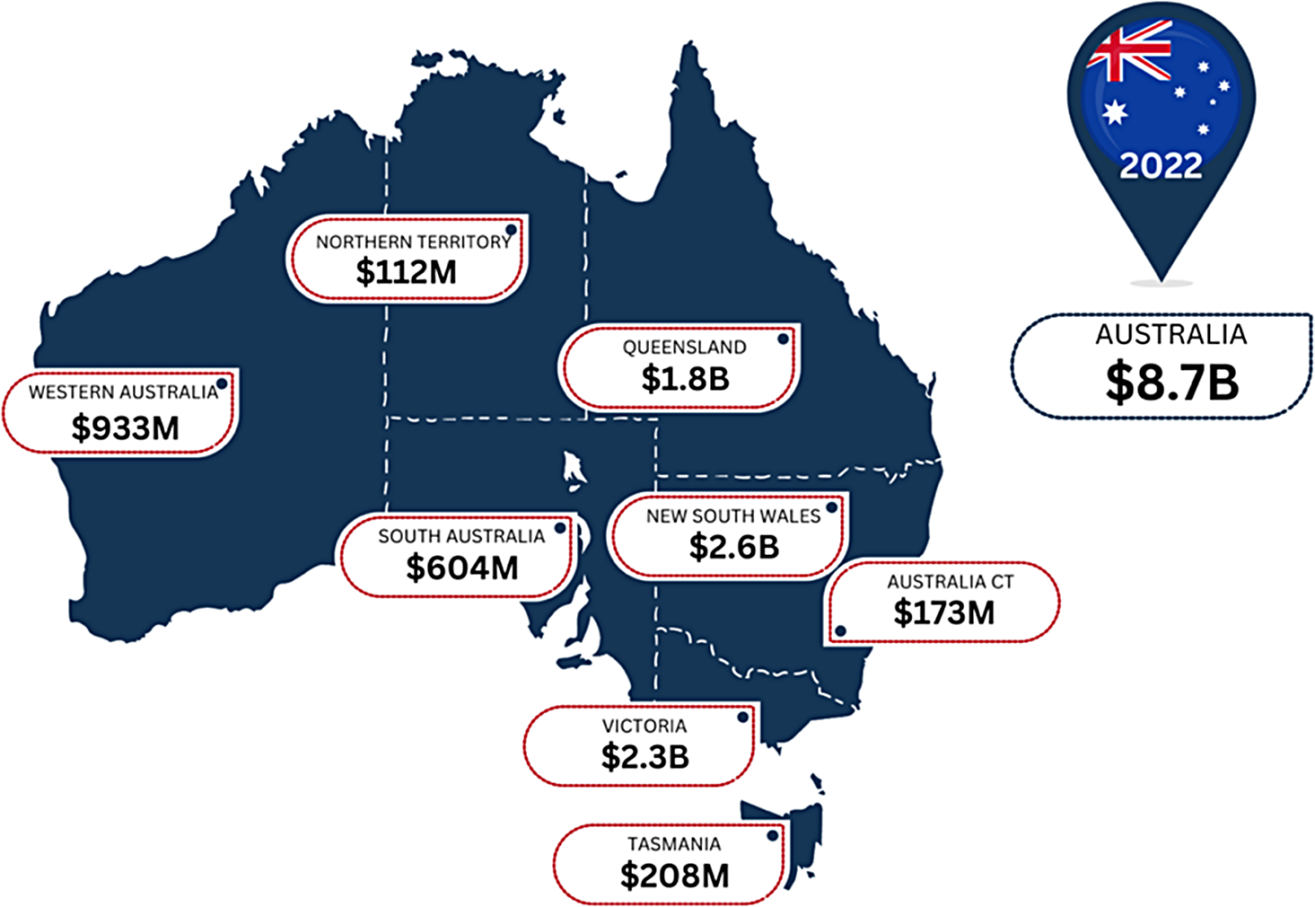
Estimated costs of wound care within Australia by state (2022).

Australia efficiently tracks its healthcare spending state by state. Several research groups have published cost related studies,^25^, ^26^ which includes a national estimate of wound care costs.^27^ None of these studies provided a regional picture of wound care expenditure. A regional analysis using our estimation model (Figure 8), presents how this is broken down across the country.

## 4 CONCLUSIONS

Comprehending the economic impact of wound care offers valuable insights to policymakers and healthcare leaders, shedding light on the broader economic implications of wound management and its costs to the payors. This knowledge serves as a foundation for informed decision‐making and the development of policies and research direction that support effective wound prevention and care practices. The impact can be both regionally or nationally influenced so a deeper understanding of regional spend can provide more directive guidance versus a national picture of costs.
